# Comparison of Growth and the Cytokines Induced by Pathogenic *Yersinia enterocolitica* Bio-Serotypes 3/O: 3 and 2/O: 9

**DOI:** 10.3389/fcimb.2017.00158

**Published:** 2017-05-01

**Authors:** Haoshu Yang, Wenpeng Gu, Haiyan Qiu, Guixiang Sun, Junrong Liang, Kewei Li, Yuchun Xiao, Ran Duan, Huaiqi Jing, Xin Wang

**Affiliations:** ^1^National Institute for Communicable Disease Control and Prevention, Chinese Center for Disease Control and Prevention, State Key Laboratory for Infectious Disease Prevention and Control, Collaborative Innovation Center for Diagnosis and Treatment of Infectious DiseasesBeijing, China; ^2^Lianyungang Center of Disease Control and PreventionLianyungang, China; ^3^Yunnan Provincial Centres for Disease Control and PreventionKunming, China; ^4^Department of Public Health, Xuzhou Medical CollegeXuzhou, China

**Keywords:** *Yersinia enterocolitica*, competitive inhibition, cytokines, bio-serotypes 3/O: 3, bio-serotypes 2/O: 9

## Abstract

Pathogenic *Yersinia enterocolitica* is widely distributed in China where the primary bio-serotypes are 3/O: 3 and 2/O: 9. Recently, the distribution of 2/O: 9 strains are being gradually replaced by 3/O: 3 strains where presently 3/O: 3 strains are the major pathogenic *Y. enterocolitica* in China. To identify the growth conditions and cytokines induced by *Y. enterocolitica* and providing some clues for this shift, we performed competitive growth *in vitro* and *in vivo* for these two bio-serotype strains; and we also compared the cytokines induced by them in infected BALB/C mice. We found 2/O: 9 strains grew more *in vitro*, while 3/O: 3 strains grew more *in vivo* regardless of using single cultures or mixed cultures. The cytokines induced by the two strains were similar: interleukin-6 (IL-6), IL-9, IL-13, granulocyte colony-stimulating factor (G-CSF), chemokines (KC), monocyte chemotactic protein 1 (MCP-1), macrophage inflammation protein-1α (MIP-1α), tumor necrosis factor-α (TNF-α), and RANTES were statistically up-regulated upon activation of normal T cells compared to the control. The cytokine values were higher in mixed infections than in single infections except for IL-6, G-CSF, and KC. The data illustrated the different growth of pathogenic *Y. enterocolitica* bio-serotype 3/O: 3 and 2/O: 9 *in vitro* and *in vivo*, and the cytokine changes induced by the two strains in infected BALB/C mice. The growth comparisons of two strains maybe reflect the higher pathogenic ability or resistance to host immune response for *Y. enterocolitica* bio-serotype 3/O: 3 and maybe it as one of the reason for bacteria shift.

## Introduction

*Yersinia enterocolitica* is widely distributed in the natural world; and being one of the few intestinal bacteria that can grow at low temperatures, the animal hosts of *Y. enterocolitica* are widely distributed, including livestock, poultry, rodents, reptiles, and aquatic animals. It causes gastrointestinal symptoms and systemic disease, e.g., liver and spleen abscess (Thoerner et al., 2003), reactive arthritis, erythema nodosum, and anemic goiter (Heyma et al., 1986; Stuart and Woodward, 1992; Gaede and Heesemann, 1995; Wang et al., 2012); and can cause septicemia and death (Bottone, 1999). Biotyping and serotyping are common and valuable methods for *Y. enterocolitica* identification where at present *Y. enterocolitica* is divided into six biotypes (1A, 1B, 2, 3, 4, and 5) and 60 serotypes. The highly pathogenic *Y. enterocolitica* bioserotype 1B/O:8 strain was distributed worldwide (Wang et al., 2008, 2010, 2012; Liang et al., 2012); while the other bio-serotypes were the lower or non-pathogenic strains and distributed all over the world (Wang et al., 2011). From our previous investigation, 58 serotypes of *Y. enterocolitica* had been found and no highly 1B/O: 8 strain has been isolated in China (Wang et al., 2008; Gu et al., 2012). The bio-serotype 3/O: 3 and 2/O: 9 strains were the primary types found in China.

Since the 1990s, the isolation numbers of bio-serotype 2/O: 9 strains have decreased, while the 3/O: 3 strains became the dominant bio-serotype in China. From 2009 to 2011 in a national survey for *Y. enterocolitica*, 2/O: 9 almost disappeared where only six O: 9 strains were identified from 862 *Y. enterocolitica* (Liang et al., 2012). The mechanisms of this change were not known, and therefore to determine the reason, we performed comparative growth of the two strains *in vitro* and *in vivo*, and compared the bacteria produced cytokine changes of the two strains in infected BALB/C mice.

## Materials and methods

### Bacterial strains

NX1998-SA98-837 (bio-serotype 3/O: 3, strain A) and NX1998-SA98-835 (bio-serotype 2/O: 9, strain B) were selected; both strains were isolated from Ningxia Province in 1998. We chose additional two pathogenic strains BJ2009-3-248 (3/O: 3, strain C) and BJ2009-3-247 (2/O: 9, strain D) isolated from Beijing in 2009. All four strains carried *ail, ystA, virF*, and *yadA* virulence genes, indicated that the strains were pathogenic ones.

### *In vitro* cultures

Two colonies of strain A and B were selected either as a single culture, or the strains were mixed as mixed cultures. All the cultures were inoculated in BHI broth and incubated at 25°C for 48 h, and shaken at 120 rpm. Colony counts were performed every 3 h, and each colony in the mixed bacterial culture was identified using monoclonal antibodies grown in our laboratory; and finally comparative growth curves were drawn. The experimental procedure was performed twice for each strain; and strains C and D were also included.

### *In vivo* cultures

Six week-old healthy female BALB/C mice were purchased from the Chinese Academy of Food and Drug Testing Laboratory Animal Resource Center. Experiments were performed using the four strains as mentioned above. For each experiment, O: 3 and O: 9 strains were mixed to infect animals using equal number of each bacterium in the experimental groups; O: 3 and O: 9 strains singly infected mice were the two control groups (O: 3 control group and O: 9 control group), and healthy mice were selected as a blank group. Approximately 10^7^ cfu/ml of the bacteria were used for intraperitoneal injection, three mice were removed randomly at 3, 6, 9, 12, 15, 18, 21, 24, 27, 30, 33, 36, 39, 42, 45, and 48 h after infection and sub-ocular sinus blood was separated and the serum was stored at −70°C. Mice were sacrificed by cervical dislocation; the spleens were removed after immersion disinfection. Three spleens were mixed with 3 ml PBS to form a suspension, and diluted 10-fold to 10^−3^; and the appropriate concentration to coat plates at 3 h was made. The plates were cultured at 25°C for 24 h.

### Cytokines

The content of cytokines from each group of mice was measured and the serum levels of IL-1α, IL-1β, IL-2, IL-3, IL-4, IL-5, IL-6, IL-9, IL-10, IL-12p40, IL-12p70, IL-13, IL-17α, eotaxin, G-CSF, GM-CSF, IFN-r, KC, MCP -1, MIP-1α, MIP-1β, and RANTES were determined using a Bio-Plex mouse 23 cytokine kit purchased from the BioRad Company (USA). TNF-α was measured using an ELISA kit purchased from the R&D Company (USA). After processing the data, a comparison of the cytokine levels in the three groups of mice was performed.

### Statistical analysis

The amount of bacteria at each time point was bacterial counts per milliliter and each cytokine measured value were expressed as mean ± standard deviation. The statistical differences between each group were analyzed using analysis of variance or the *T*-test where differences had significance when *P* ≤ 0.05.

### Ethics statement

The animals were handled according to the national criterion for animal investigation of China (Ethics Review Committee [Institutional Review Board (IRB)] of National Institute for Communicable Disease Control and Prevention, Chinese Center for Disease Control and Prevention). The experimental and detection protocols were carried out in accordance with relevant guidelines and regulations. The ethics of this study was approved by Ethics Review Committee of National Institute for Communicable Disease Control and Prevention, Chinese Center for Disease Control and Prevention. The License numbers were ICDC-2015001 and ICDC-2015002.

## Results

The initial bacteria concentration of NX1998-SA98-837 was 1.03 × 10^8^ cfu/ml and NX1998-SA98-835 was 0.89 × 10^8^ cfu/ml; and BJ2009-3-248 was 0.65 × 10^8^ cfu/ml and BJ2009-3-247 was 0.87 × 10^8^ cfu/ml.

**Figure 1:**
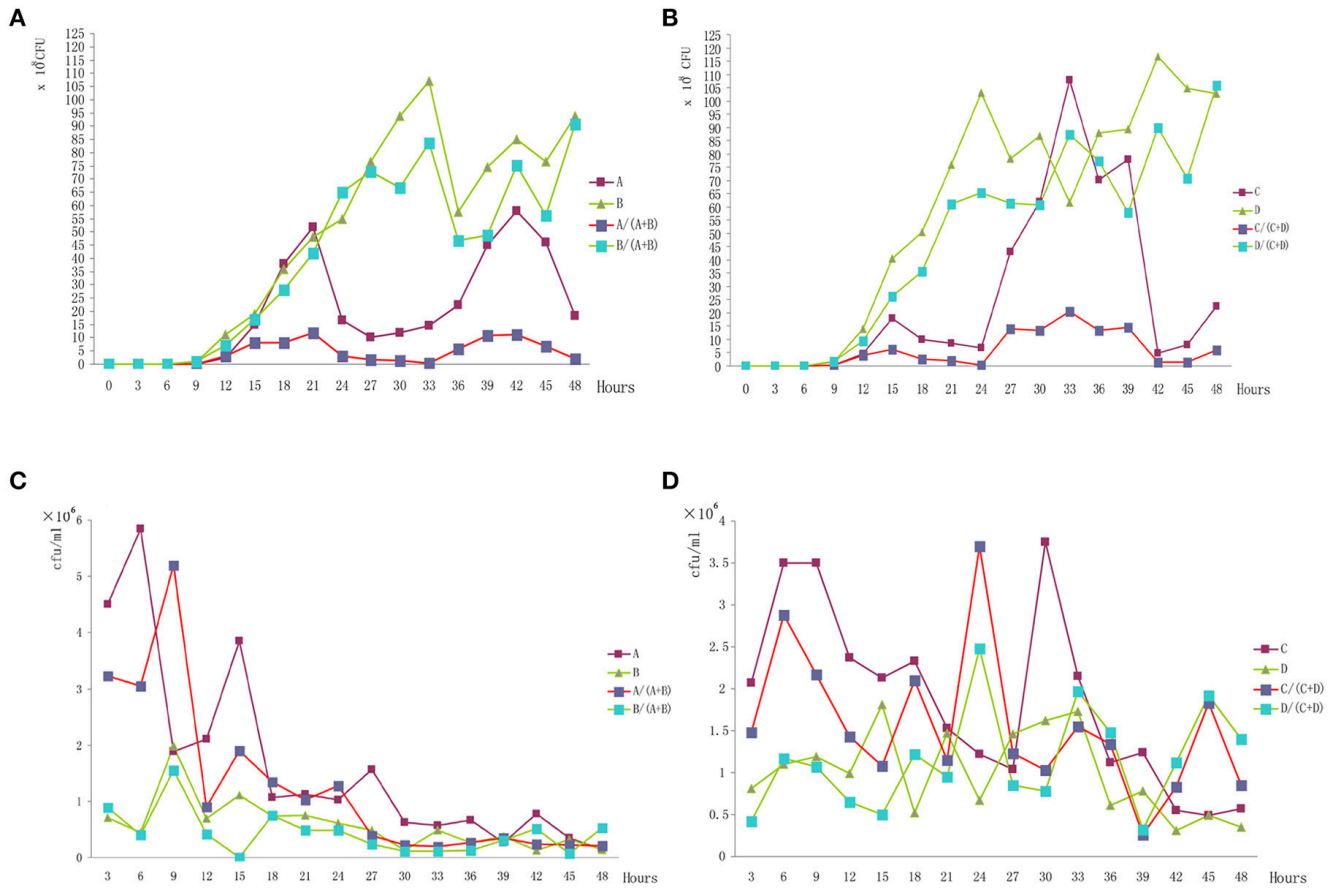
**Bacterial numbers of each strain ***in vitro*** and ***in vivo***. (A)** The growth curves of Ningxia strains *in vitro*. **(B)** The growth curves of Beijing strains *in vitro*. **(C)** The growth curves of Ningxia strains *in vivo*. **(D)** The growth curves of Beijing strains *in vivo*.

The growth of different bio-serotype strains *in vitro* or *in vivo* was different. Comparing the growth curves of the four strains *in vitro*, we found that 2/O: 9 strains had statistically significant higher growth than 3/O: 3 strains (Figures 1A,B), either in single culture or mixed cultures. However, the growth curves of four strains *in vivo* were inverse, 3/O: 3 grew statistically significantly higher than the 2/O: 9 (Figures 1C,D), either in single infection or mixed infection.

**Figure 2:**
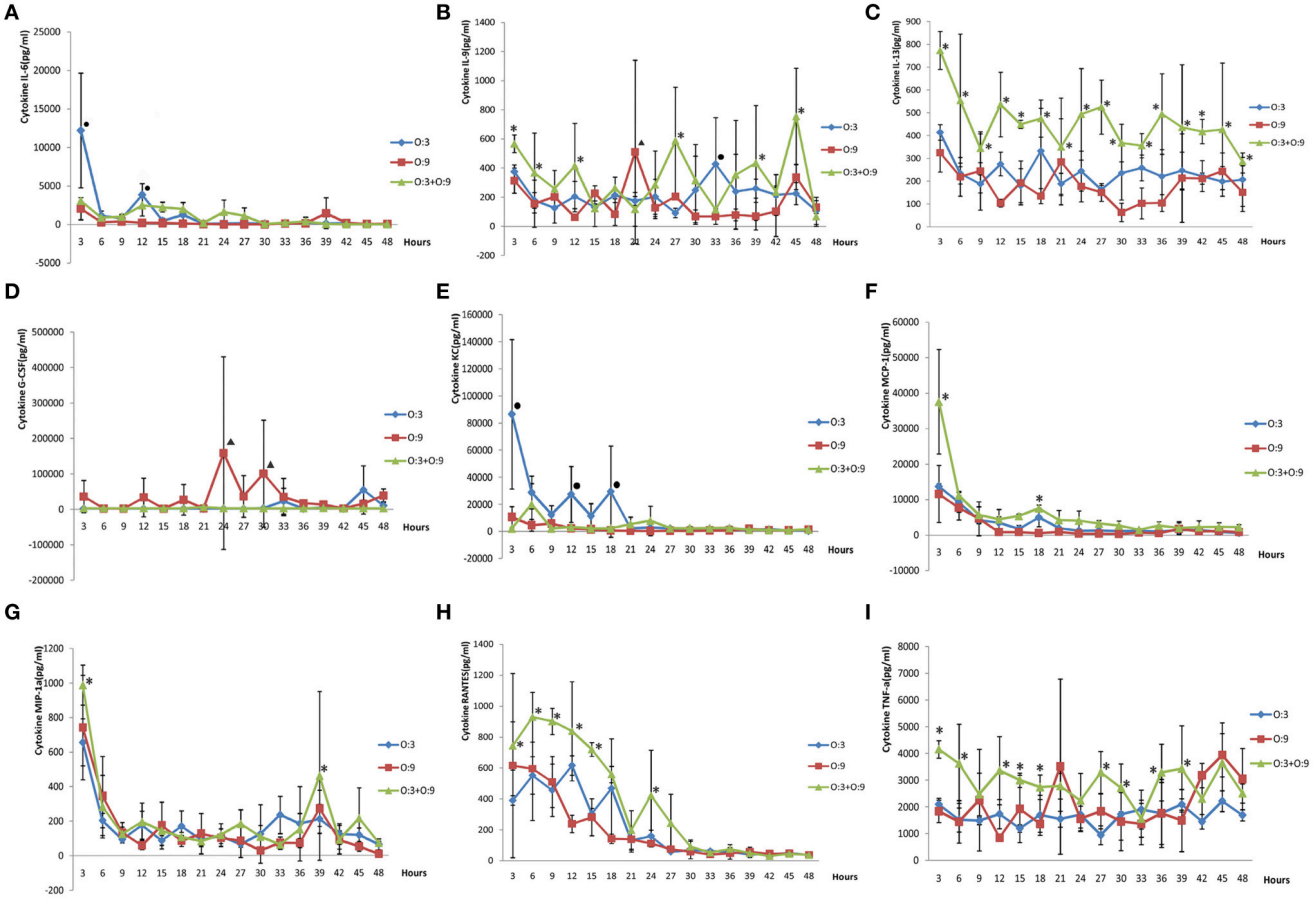
**The cytokine changes induced by two bio-serotype strains in BALB/C mice. (A)** IL-6, **(B)** IL-8, **(C)** IL-13, **(D)** G-CSF, **(E)** KC, **(F)** MCP-1, **(G)** MIP-1α, **(H)** RANTES, I: TNF-α. ^*^The cytokines with mixed infections were statistically different compared to other cytokine values. •The cytokines of 3/O: 3 infections were statistically different compared to other cytokine values. ▴ The cytokines of 2/O: 9 infections were statistically different compared to other cytokine values.

The cytokine changes in mouse sera infected with the two bio-serotype strains were similar where only IL-6, IL-8, IL-13, G-CSF, KC, MCP-1, MIP-1α, RANTES, and TNF-α were different statistically compared to the control group (Figure 2). For IL-8, IL-13, MCP-1, MIP-1α, RANTES, and TNF-α, the cytokine values using the mixed infection with two strains were higher than single infections; and there was no difference with 3/O: 3 or 2/O: 9 independently infected. For IL-6 and KC, the values from 3/O: 3 infections were higher than the 2/O: 9 infections and mixed infections; and for G-CSF, the values of 2/O: 9 infections were higher than 3/O: 3 and mixed infections. The experiments were repeated twice, and the all the results were consist.

## Discussion

In our study, the competitive growth of the two bio-serotype strains were totally different, the 3/O: 3 grew greater than 2/O: 9 *in vivo*; conversely, the 2/O: 9 grew greater than 3/O: 3 *in vitro*. This suggested the 2/O: 9 grew better without a host; however, the 3/O: 3 had stronger resistance to the defense mechanisms of the hosts. Therefore, the clearance of the 3/O: 3 was slower than the 2/O: 9 in BALB/C mice; and might reflected the different pathogenic abilities. This showed the higher resistant ability of 3/O: 3 to host clearance than the 2/O: 9, and might be a possible explanation for distribution of the two strains in human hosts.

Cytokines participate in cellular immunity, humoral immunity, hematogenesis regulation, cell proliferation, and injury repair; however, excess cytokine secretion leads to immunopathogenic effects. Some research showed excess cytokines induced host death after infections (Hancock et al., 1986; Segura et al., 1999; Smith et al., 2008). Currently, the studies on different serotypes of *Y. enterocolitica* that cause different secretions of cytokines were shown (McNally et al., 2006). In our study here, the *Yersinia* highly susceptible BALB/C mice were used instead of cells (Autenrieth and Heesemann, 1992). Cytokines involved in this study, IL-6, IL-8, MCP-1, MIP-1α, and RANTES in a relatively short period of time reach their peaks in both the two control groups and the experimental group. These cytokines were inflammatory cytokines. This was consistent with the reported literature: *Yersinia* infection of epithelium (HeLa) cells can significantly enhance the levels of transcription and secretion of inflammatory cytokines MCP-1, GM-CSF, and TNF-α. One hour after infection transcript levels of these cytokines began to increase and within 3–4 h reached their peak and then gradually decline (Kampik et al., 2000). These cytokines played important roles in the hosts' immune response against *Y. enterocolitica* and the cytokine changes for *Y. enterocolitica* infection were similar using cell infected models or animal infected models.

Previous studies showed the cytokine values of highly pathogenic *Y. enterocolitica* were higher than the lower pathogenic strains (Gu et al., 2013). Brucella and 2/O: 9 bio-serotype *Y. enterocolitica* caused different cytokine changes, involving their different pathogenic ability and life cycles (Wang et al., 2013). However, in our study lower pathogenic *Y. enterocolitica* had similar cytokine changes compared with 3/O: 3 and 2/O: 9 strains. Therefore, the similar cytokine changes were caused by analogical pathogenic ability of the *Y. enterocolitica* strains. The different pathogenic abilities or life cycles were important for cytokine changes in *Y. enterocolitica* infections. The cytokines induced by two kinds of strains were similar, while the growth conditions were different; maybe this reflected the different pathogenic ability or defense ability to hosts, and it may as one of the reason for bacteria shift.

## Author contributions

Design of the work: HJ and XW. Do the experiments: HY, HQ, JL, KL, YX, and RD. Analysis the data: HY and WG. Drafting the work: WG. Revising it critically for important intellectual content: GS.

## Funding

This work was supported by the National Sci-Tech Key Project (2012ZX10004-201, 2012ZX10004-212, and 2013ZX10004203-002).

### Conflict of interest statement

The authors declare that the research was conducted in the absence of any commercial or financial relationships that could be construed as a potential conflict of interest.
